# Integration of CLIP experiments of RNA-binding proteins: a novel approach to predict context-dependent splicing factors from transcriptomic data

**DOI:** 10.1186/s12864-019-5900-1

**Published:** 2019-06-25

**Authors:** Fernando Carazo, Marian Gimeno, Juan A. Ferrer-Bonsoms, Angel Rubio

**Affiliations:** Tecnun (University of Navarra), Paseo Manuel Lardizábal 15, 20018 San Sebastián, Spain

**Keywords:** Alternative splicing, Splicing factor, RNA-binding protein, RNA-seq, CLIP-seq

## Abstract

**Background:**

Splicing is a genetic process that has important implications in several diseases including cancer. Deciphering the complex rules of splicing regulation is crucial to understand and treat splicing-related diseases. Splicing factors and other RNA-binding proteins (RBPs) play a key role in the regulation of splicing. The specific binding sites of an RBP can be measured using CLIP experiments. However, to unveil which RBPs regulate a condition, it is necessary to have a priori hypotheses, as a single CLIP experiment targets a single protein.

**Results:**

In this work, we present a novel methodology to predict context-specific splicing factors from transcriptomic data. For this, we systematically collect, integrate and analyze more than 900 CLIP experiments stored in four CLIP databases: POSTAR2, CLIPdb, DoRiNA and StarBase. The analysis of these experiments shows the strong coherence between the binding sites of RBPs of similar families. Augmenting this information with expression changes, we are able to correctly predict the splicing factors that regulate splicing in two gold-standard experiments in which specific splicing factors are knocked-down.

**Conclusions:**

The methodology presented in this study allows the prediction of active splicing factors in either cancer or any other condition by only using the information of transcript expression. This approach opens a wide range of possible studies to understand the splicing regulation of different conditions. A tutorial with the source code and databases is available at https://gitlab.com/fcarazo.m/sfprediction.

**Electronic supplementary material:**

The online version of this article (10.1186/s12864-019-5900-1) contains supplementary material, which is available to authorized users.

## Background

The expansive diversity of the transcriptome – induced by pre-mRNA splicing-plays a key role in the development of a broad spectrum of human diseases [1–3]. Specifically, all the hallmarks of cancer (such as angiogenesis, cell immortality, avoiding immune system response, etc.) have a counterpart in aberrant splicing of key genes [4, 5].

RNA-binding proteins (RBPs) bind to single-or double-stranded RNA and conduct post-transcriptional modifications of pre-mRNA (alternative splicing, mRNA stabilization, mRNA location, polyadenylation, translation, etc.) [6]. RBPs that regulate mRNA splicing are called splicing factors. Changes in splicing factors–such as mutations or expression changes– directly affect splicing and may result in the expression of less standard isoforms that, in turn, results in an anomalous gain or loss of protein function [7].

The link between RBPs and splicing has been studied “in-silico” by analyzing RNA binding motifs of RBPs, as reviewed in [8]. RBPs’ binding motifs are usually represented by position weighted matrices (PWMs) that provide the probability of having a specific nucleotide in each motif’s position. PWMs are gathered from different databases [9–13] and scanned into the genome to find putative binding sites. The weakest step of this pipeline is the identification of the specific binding sites for the RBPs. PWMs are usually short (>40% PWMs are shorter than 7nt) and provide low specificity [8]. This precludes hits that are statistically significant. Implying that binding rules are diffuse (at least, on the PWM level), binding is probably co-defined by contextual information.

RBP–RNA interactions can be also experimentally identified [14] by employing cross-linking and immunoprecipitation (CLIP) coupled with high-throughput sequencing. CLIP experiments are more suitable to uncover the binding sites of a specific RBP than scanning its binding motifs, since they return the real binding sites of a protein rather than the predictions of a motif-scanning algorithm [8]. A CLIP experiment targets a specific protein. In many cases this protein is not known beforehand and the researcher, based on his/her expertise, must decide which are the “suspects” to run the CLIP experiments against. A methodology for predicting active splicing factors would be desirable to help the researcher select specific RBPs candidates before conducting any CLIP experiment.

Previous works to predict active splicing factors have used PWMs instead of CLIP experiments [1, 7, 15–20]. In addition, most of these references are implemented on a case-by-case basis, which implies that these pipelines are only capable of predicting a few splicing factors, instead of a large group of them. To our knowledge, there is no methodology for predicting splicing factors using the information of CLIP experiments.

In this work, we systematically collect, integrate and analyze 937 CLIP experiments stored in four well-known CLIP databases: POSTAR2 [21], CLIPdb [22], DoRiNA [23] and StarBase [24]. Using this information, we present a methodology for predicting context-specific splicing factors based on CLIP experiments and RNA-seq. This pipeline relates splicing factor binding sites –obtained from the CLIP databases- with the splicing events that show differential usage across the conditions. Using a GSEA-like enrichment analysis, we estimate the potential splicing factors that conduct splicing in the studied condition. Combining this information with expression changes, we were able to correctly predict the knocked-down splicing factors in several gold-standard experiments. Comparing this methodology with previous approaches, we found that the ranks of splicing factors that affect each condition were systematically higher and more significant using CLIP experiments than using PWMs. The whole pipeline is ready to use with any RNA-seq experiment.

## Results

### A unified database of human and mouse CLIP experiments

We downloaded and integrated the CLIP experiments contained in POSTAR2, CLIPdb, DoRiNA and StarBase databases as described in the Methods section. Five of these experiments were discarded from the analysis because the RBPs under study were mutated. The information of the CLIP experiments can be found in the Additional file 1 Supplementary material S1.

**Fig. 1:**
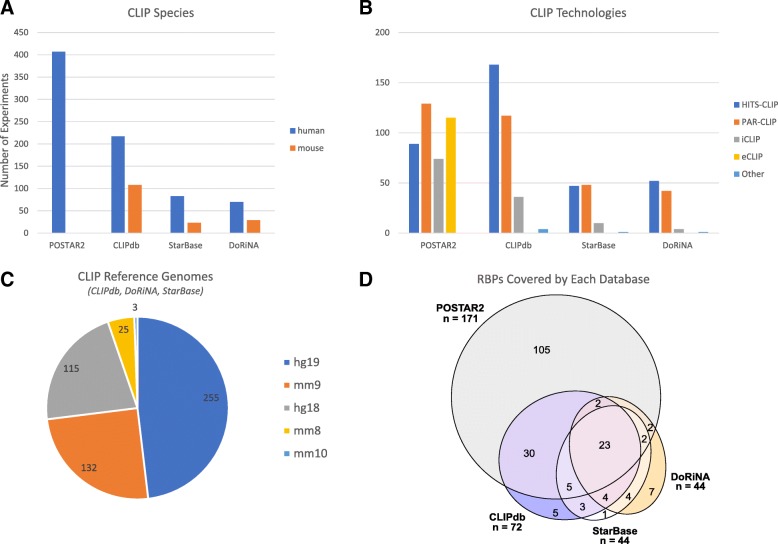
Overview of the four databases integrated in this work. **a** Number of experiments per organism in each database. **b** Number of experiments grouped by technology. **c** Different genome versions available in the databases. All the experiments in POSTAR2 correspond to human hg38. **d** Proportional Venn diagram - built using [25] - of the RBPs covered by each database. The four databases cover 195 different RNA-binding proteins (RBPs). The basis of CLIP database is POSTAR2 (171 RBPs), comprising all but 24 other RBPs. However, other databases add up a significant part of experiments (Additional file 1 Supplementary material S1)

CLIPdb, DoRiNA and StarBase include CLIP experiments from different species (e.g. human, mouse, fly, worm, etc.). In contrast, POSTAR2 only contains human CLIP experiments. We included both human and mouse CLIP data. Overall in this work 937 CLIP experiments have been integrated (70% human and 30% mouse) (Fig. 1).

In total, 87% CLIP experiments (816 out of 937) belong to the three main CLIP technologies: HITS-CLIP (38%), PAR-CLIP (36%) and iCLIP (13%) (Fig. 1b). Interestingly, POSTAR2 incorporates 115 eCLIP experiments, while the other databases lack this technology. An important proportion of these CLIP experiments (17%) targeted *AGO2*. POSTAR2 is the biggest database with 43% of the total CLIP experiments. CLIPdb includes 34% experiments and DoRiNA and StarBase around 18% each one. CLIP experiments arise from different species and reference genomes (Fig. 1c). POSTAR2’s CLIP experiments were lifted over to hg38 by POSTAR2’s authors [21]. Regarding CLIPdb, DoRiNA and StarBase, almost 50% of the reference genomes belong to hg19. The rest of them correspond to mm9 (25%), hg18 (22%), mm8 (5%) and mm10 (1%).

The 937 downloaded CLIP experiments cover 195 different RBPs (Additional file 1 Supplementary material S2). The most complete database is POSTAR2 which collects 171 RBPs, followed by CLIPdb with 72. POSTAR2 and CLIPdb share a large number of RBPs (*n* = 30), since POSTAR2 integrated the human CLIP experiments of CLIPdb. All these CLIP experiments were converted to human hg38 genome’s version, so that they can be compared.

### RBPs binding sites are coherent with protein families

RBPs regulate splicing events by binding to regions near to the alternative exons -typically 300–400nt upstream and downstream the alternative exons [26]. We identified 118,830 possible splicing events in Gencode v24 (hg38) [27] using EventPointer [28]. We extracted the adjacent splicing regions of these events by selecting 400 nt upstream and downstream the alternative exons (Fig. 4, Panel 1).

The CLIP files, previously converted to hg38, were mapped against adjacent splicing regions. For each RBP, we summarized its CLIP experiments into a single dataset following an inclusive criterion: if a binding site is annotated to any CLIP experiment, it is considered as a putative regulation. As a result of this mapping, we got a binary matrix (named ExS, Events x Splicing factors) relating splicing events with RBPs (Fig. 4, Panel 2). This matrix –as an RData file- is available in the GitLab repository.

**Fig. 2:**
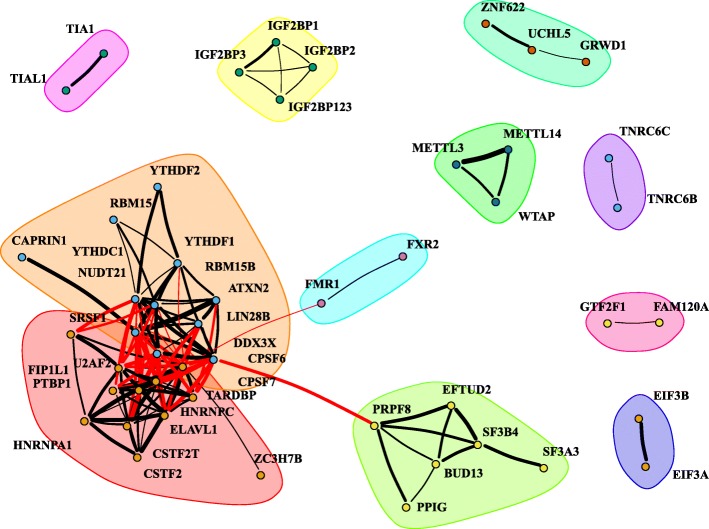
CLIP similarities of RNA-binding proteins (RBPs). A line connecting two RBPs represents a Pearson correlation > = 0.46. The width of the line is proportional to the correlation value. RBPs are grouped in clusters. Links corresponding to RBPs in different clusters are represented as red lines

Using the columns of the ExS matrix, it is possible to evaluate how similar the binding sites of different RBPs are. We computed the Pearson correlation between every pair of RBPs and assigned a statistical significance to such relationship with a Fisher’s exact test (Additional file 2: Figure S3). Pairs of RBPs with high correlation bind to similar splicing events. Using this information, we also built a similarity network of RBPs by setting two thresholds in the Pearson correlation *r*_*s*_ ≥ 0.46 and FDR < 0.1 (Fig. 2). Remarkably, it shows that RBPs that belong to similar families tend to cluster together (Fig. 2; e.g. *IGF2BP-X* (yellow cluster)*, METTL-X* (dark-green cluster)*, SF3-X* (green cluster)*, TRNC-X* (purple cluster), *YTHD-X* (orange cluster)). Interestingly, some clusters of RBPs connect different families of proteins (e.g. the *SF3-X* cluster contains *EFTUD2* and BUD13). This means that those RBPs could be interacting within the spliceosome. For instance, *SF3B4* and *SF3A3* are constituents of the U2 snRNP [29]. In turn, *PRPF8* and *EFTUD2* form the U5 snRNP, which interacts with U2 snRNP within the spliceosome [30].

### Accurate prediction of context-specific splicing factors

We have developed a methodology to suggest the splicing factors that are the major conductors of splicing in a condition, by using the relationships between CLIP datasets (i.e. the real binding sites of RBPs) and transcript expression. To test this approach, we selected two datasets that knock-down different RBPs, so that we have gold-standard RBPs. In the first dataset, the splicing factor *SRSF1* was knocked-down using siRNA on the A549 lung adenocarcinoma cell line [31]. The second dataset individually depleted three RBPs implicated in amyotrophic lateral sclerosis: *FUS*, *TAF15* and *TARDBP* [32]. The experiment was performed in human iPSCs derived from dermal fibroblast cells of a healthy individual. The four knock-down RBPs of both datasets will be referred to as: *KD-SRSF1*, *KD-FUS, KD-TAF15* and *KD-TARDBP*. In these experiments, we a priori know which are the splicing factors that ultimately change the splicing patterns (i.e. the depleted ones).

For each dataset, we estimated the Percent Spliced-In (PSI) of the 118,830 putative events and calculated which events show differential splicing in each knock-down by using Event Pointer (See Methods section for more details). Then -with the aid of the ExS matrix- we compared the RBPs that bind against differentially spliced with non-differentially spliced events using a Fisher’s exact test. We ranked the RBPs according to the resulting *p*-value (which will be referred to as CLIP *p*-value). For the final ranking, we imposed the candidate RBPs to be differentially expressed in the conditions under study. Since there is no general-purpose methodology for predicting RBPs, we cannot compare our results with previous algorithms. Nevertheless, we have implemented the approach based on RBPs’ binding motifs (PWMs) - using the ATtRACT database [9] - and compared our results with it.

The *KD-SRSF1* experiment consists of three conditions: cells treated only with the vehicle of the transfection (Lipofectamine 2000, Invitrogen), cells treated with scramble siRNA (i.e. a sequence that will not lead to the specific degradation of any cellular mRNA) and cells transfected with a siRNA that targets SRSF1. These three groups are referred to as Control, SCR and KD-SRSF1 respectively. Each condition has three biological replicates that, in turn, are hybridized three times.

In [31] it was shown that SRSF1 was properly depleted in the samples. Before calculating the splicing events, we set a filter based on gene expression (i.e., if a gene is not expressed, there is no point in discussing its splicing). All genes whose expression was under quantile 0.25 in all the samples were discarded. Out of the theoretical 97,482 events interrogated by the array, 35,963 passed the expression threshold and 3686 showed a *p*-value < 0.001 according to the Event Pointer test (approx. 4% of the events).

Seven RBPs passed the following filters: CLIP *p*-value < 0.05; limma *p*-value < 0.05 and |log2 FC| > 0.58 (Table 1, Additional file 2: Figure S3). *SRSF1* -the knock-down gene- ranked 1st out of them with strong significance (CLIP *p*-value = 5,16E-24). Interestingly, 4 out of the other 6 RBPs of the ranking (*UCHL5, SF3A3, HNRNPD* and *EFTUD2*) have strong relationships with SRSF1 according to the STRING database [33] (PPI enrichment *p*-value: 2.57e-05). These results show the relationships and the tight coupling among RBPs in the experiments, as the depletion of *SRSF1* provokes significant changes in the expression of other RBPs. In [8] we showed that using PWMs it was possible to predict *SRSF1* as a key splicing regulator. In that study, *SRSF1* ranked 13th with smaller statistical significance (PWM *p*-value = 8.32E-4).

**Table 1:** Ranking of RNA-binding proteins (RBPs) for the experiments: KD-SRSF1, KD-FUS and KD-TARDBP (CLIP *p*-value < 0.05; limma *p*-value < 0.05; |log2 FC| > 0.58). Four groups of columns are separated by thick vertical black lines are shown: i) knock down (KD) genes and RBP of the ranking; ii) the prediction using the pipeline presented in this work (CLIP experiments); iii) differential expression (knock-down vs normal) and iv) the same prediction using previous algorithms based on RBPs’ consensus binding motifs –represented as Position Weighted Matrices (PWMs). NA: the PWM is not available for this RBP. N.S.: non-significant

Experiment	RBP	Ranking by CLIP *p*-value (out of 195)	Differentially spliced hits (Expected)	Differentially spliced hits (Found)	CLIP *p*-value (Fisher)	Expression Fold change (log2)	limma adjusted *p*-value	Ranking by PWM *p*-value (out of 123)	PWM *p*-value
KD-SRSF1	***SRSF1***	**10**	**396**	**620**	**5 .16E-24**	**−1 .54**	**9 .18E-29**	**13**	**8.32E-04**
	*LIN28B*	20	561	747	3 .75E−15	0 .96	7 .76E-14	NA	NA
	*UCHL5*	27	261	384	1 .03E-13	-1 .52	7 .61E-21	NA	NA
	*SF3A3*	37	266	324	7 .03E-11	−0 .83	2 .77E-22	NA	NA
	*HNRNPD*	46	69	164	2 .94E− 08	−0 .63	1 .49E-16	15	1 .11E−03
	*EFTUD2*	62	454	520	1 .29E-05	-0 .66	2 .69E−19	NA	NA
	*TAF15*	115	83	149	4 .75E− 02	−0 .78	5 .05E-17	NA	NA
KD-FUS	***FUS***	**11**	**375**	**465**	**2 .35E−09**	**-1 .09**	**9 .77E− 02**	**112**	**N.S.**
	*LIN28A*	25	163	219	1 .78E− 06	0 .69	1 .43E-01	1	9 .54E-02
	*FBL*	56	49	73	3 .12E-04	-0 .81	3 .10E-01	NA	NA
	*YBX3*	64	145	182	4 .94E-04	-0 .79	2 .08E-01	NA	NA
	*CPSF2*	109	40	52	2 .58E-02	-0 .70	9 .91E-02	NA	NA
KD-TARDBP	***TARDBP***	**20**	**688**	**746**	**2 .10E-05**	**-0 .87**	**2 .16E-02**	**13**	**1 .30E-02**
	*RBM22*	26	160	205	6 .69E-05	0 .64	1 .93E-02	NA	NA
	*PTBP2*	58	144	174	3 .74E-03	-0 .60	4 .57E-02	NA	NA
	*SF3A3*	68	223	255	7 .86E-03	0 .64	7 .83E-02	NA	NA
	*FBL*	81	49	63	1 .81E-02	0 .89	1 .14E-01	NA	NA
	*RBP*	83	37	49	1 .92E-02	0 .68	1 .69E-02	NA	NA

The second experiment inhibited three RBPs: *KD-FUS, KD-TAF15* and *KD-TARDBP* [32]. This dataset contains RNA-seq data of five conditions: scramble siRNA, cells transfected with siRNAs that individually target *FUS, TAF15* and *TARDBP* and a double depletion of *FUS* and *TAF15*. In our analysis, we consider the three individual knock-down samples and the scramble, which will be referred to as *KD-FUS*, *KD-TAF15*, *KD-TARDBP* and SCR.

Transcripts and genes expression were estimated from RNA-seq data using the standard pipeline of Kallisto [34]. Gencode v24 (hg38) was chosen as the reference transcriptome [27]. This transcriptome contains 199,169 transcripts and 58,684 genes. As a preliminary step, we compared gene expression changes between conditions SCR, *KD-FUS*, *KD-TAF15* and *KD-TARDBP* in order to confirm the knock-down effect of the inhibitions. As expected, *FUS*, *TAF15* and *TARDBP* were under-expressed in the knock-down samples. Interestingly, as it happened in the *KD-SRSF1* experiment, other RBPs also significantly changed their expression, which underlines the strong interactions between RBPs.

We then compared the splicing events of each condition against SCR. For this task, we modified Event Pointer to identify splicing events using transcript expression. We set an expression filter to remove lowly expressed splicing events (see methods for more information). Out of the theoretical 118,830 events of GenCode v24, 80,747 passed the expression threshold and 1791 (*KD-TARDBP)*, 1004 (*KD-FUS*) and 945 (*KD-TAF15*) showed a *p*-value < 0.001 according to the Event Pointer test.

In the experiments *KD-FUS* and *KD-TARDBP*, 5 and 6 RBPs were predicted to be putative splicing regulators respectively (CLIP *p*-value < 0.05; limma *p*-value < 0.05; |log2 FC| > 0.58). In both experiments, the knock-down RBP -*FUS* and *TARDBP*- had the best CLIP *p*-value (CLIP *p*-value 2.35E-09 and 2.10E-05 respectively), which stresses the ability of using CLIP experiments to decipher the regulation rules of alternative splicing (Table 1).

In the *KD-TAF15* condition, no RBP was predicted to be a splicing regulator (CLIP *p*-value < 0.05; limma *p*-value < 0.05; |log2 FC| > 0.58). These findings agree with the original paper as it highlights the low influence of *TAF15* in alternative splicing [32].

If the RBPs expression is not used as complementary information to build the ranking (i.e. only the CLIP information is used to make the prediction), the ranking of the knock-down RBPs drops some positions (*SRSF1* = 10th; *FUS* = 11th; *TARDBP* = 20th). The reason for this is that some RBPs, which usually belong to similar families, share similar binding patterns and, in turn, they have similar, or even smaller, CLIP *p*-values (Additional file 2: Figure S3).

One of the experiments under study selected *FUS*, *TAF15* and *TARDBP* (referred to as *TDP43* in the reference), since they are known to be related to amyotrophic lateral sclerosis (ALS) [32]. *DDX3*, which ranks 1st and 7th in the KD-*TARDBP* and KD-*FUS* conditions respectively, is also known to play an important role in ALS by affecting neurite outgrowth [35].

We finally implemented the PWM-based pipeline to deal with RNA-seq data and tested the prediction for KD-*FUS* and KD-*TARDBP* (*TAF15*’s binding motifs are not available in the ATtRACT database). *FUS* was non-significant using PWMs, so it was not able to be predicted. *TARDBP* ranked 13th (out of 123 RBPs, see Methods) with a smaller *p*-value than using CLIP (PWM *p*-value = 1,30E-02). When using exclusively the binding sites information, *TARDBP* ranked better using PWM than using CLIP (13th versus 20th). After filtering by expression (limma *p*-value < 0.05; |log2 FC| > 0.58), *TARDBP* ranked 3rd using PWMs.

## Discussion

In this work, we have systematically collected CLIP experiments of RBPs stored in the POSTAR2, CLIPdb, DoRiNA and StarBase databases. We have integrated them into a single genome reference (hg38). We also studied the relationships between RBPs and splicing events and shown the high coherence between the binding sites of splicing factors of similar families. In addition, we have developed a methodology for predicting context-specific splicing factors based on genome-wide CLIP experiments and RNA-seq or splicing microarrays. We have tested this methodology in four controlled experiments in which a splicing factor was depleted using siRNAs. In these experiments we were able to correctly predict the knock-down splicing factors.

We explored the relationships between the binding sites of different splicing factors by mapping CLIP binding sites against splicing regions. We highlighted the strong coherence between CLIP experiments of similar families. This fact is a consequence of the collaboration of splicing factors: several splicing factors cooperate to control the splicing of a gene [36].

Considering the prediction of RBPs, we proposed a method based on CLIP enrichment analysis of the RBP binding sites for alternatively spliced events. This method is able to narrow down the search to a few splicing factors candidates that potentially regulate splicing of an experiment. In three -KD-*SRSF1*, KD-*FUS* and KD-*TARDBP*- out of four cases, the depleted splicing factor was included in the list of candidates and had the best CLIP *p*-value among differentially expressed RBPs. In the fourth case -KD-*TAF15* experiment- no RBP was predicted to be a splicing regulator. Interestingly, *TAF15* was previously found to play a minimal role in the regulation of alternative splicing [32].

The three SRSF1, FUS and TARDBP splicing factors had strongly significant CLIP *p*-values in their corresponding experiments although other splicing factors had even lower CLIP *p*-values. The CLIP *p*-value alone is not able to distinguish between direct and indirect effects of RBPs due to the strong correlations between the binding sites of RBPs. However, the combination of CLIP enrichment analysis with differential expression of RBPs helps to reduce the list of potential splicing factors –including the true positives.

Previous approaches to predict RBPs scan the preferred binding motifs of RBPs (PWMs) in the transcriptome to find potential binding sites. The limitation of these methodologies mainly relies on scanning the binding sites, since PWMs are usually short and non-informative. Consequently, scanning them is prone to have too many potential hits in the transcriptome.

According to the ATtRACT database, more than a half of all PWMs have 7nt or less and, as we showed in [8], only motifs >7nt achieve statistical significance. In the two knock-down experiments used in this work, CLIP experiment data provide better sensitivity and specificity to predict RBPs than PWMs. Besides, 195 RBPs have CLIP experiments available, as opposed to 123 RBPs that have PWMs with significant hits in the transcriptome.

The results were encouraging: although the cell lines in the CLIP database did not match the cell lines of the experiments, it was possible to recover the depleted splicing factors. Previously, we found that this improvement also occurs combining CLIP experiments from different species [8].

With CRISPR-Cas9 being more accessible, this pipeline could also be validated knocking out a splicing factor using this technique. In this case, the statistical approach should be changed accordingly. Using CRISPR, the knocked out gene -in this case a splicing factor- does not necessarily change its expression. Therefore, the statistical part related with changes in the expression should not be included and only the statistical part related with CLIP experiments should be applied.

The standard use of this pipeline is to provide a sound hypothesis on the origin of the splicing changes in an experiment. Somehow, it is similar to studies that, by studying the transcription factors related to differentially expressed genes, provide a transcription factor that could be the potential cause of the changes. Here, instead of differential expression, we use differential alternative splicing and, instead of providing transcription factor candidates, we provide splicing factor candidates. For example, a user can compare the splicing status of normal tissue against its tumoral counterpart. This pipeline provides potential candidates of the splicing factors causing these changes.

Another potential use is to check the mediated effect of a gene in alternative splicing. In order to do that, a specific gene can be knocked down using CRISPR-Cas9. In the case the gene is not a splicing factor, the pipeline can be used to infer which are the splicing factors that are affecting the splicing patterns of the sample.

As high-throughput CLIP technologies are applied to more cells and tissue types in the near future, a larger set of splicing factors may be studied following this methodology broadening the scope of this work.

## Conclusions

In this work, we have developed a methodology to predict context-specific splicing factors based on the combination of CLIP experiments with transcriptomic data. For this task, we systematically collect, integrate and analyze 937 genome-wide CLIP experiments stored in four CLIP databases: POSTAR2, CLIPdb, DoRiNA and StarBase. This integrated database is publicly available.

Augmenting this information with expression changes, we predict the splicing factors that regulate splicing in two gold-standard experiments in which some specific splicing factors are knocked-down. The source code, databases and a tutorial to perform an equivalent analysis with other data are available in the GitLab repository, https://gitlab.com/fcarazo.m/sfprediction.

This methodology can be used to predict the active splicing factors in either cancer or in any other condition with the only information of transcript expression.

## Methods

**Fig. 3:**
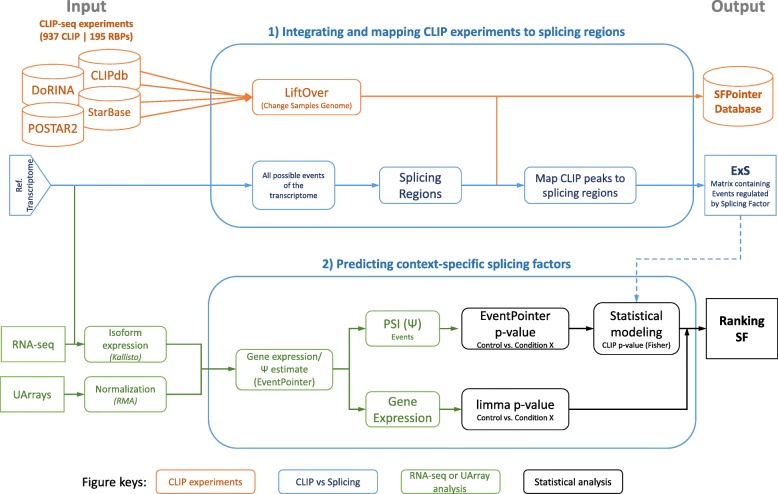
Overview of the two main pipelines in this work: 1) Integration and mapping CLIP experiments to splicing regions and 2) predicting context-specific splicing factors. The tasks done in both pipelines are represented by colors: identifying the transcriptome binding sites of RBPs using previous CLIP experiments (orange); construction of a matrix containing information of specific events for each splicing factor (blue); calculating alternative splicing events from RNA-seq data or microarray data (green); and combining both results with a statistical pipeline to obtain a ranking of splicing factors (black)

We have developed and integrated two main pipelines (Fig. 3): integrating and mapping CLIP experiments to splicing regions and predicting context-specific splicing factors using CLIP experiments.

The output of the first pipeline is twofold. It consists of an integrated database of splicing factors binding sites mapped to the hg38 version of the human genome and the correspondence of these sites with annotated splicing events of the GenCode 24 version of the human transcriptome. We represented this correspondence as an indicial matrix, which will be referred as ExS (Events x Splicing factors).

The second pipeline takes as input the ExS matrix and the expression of splicing events calculated using either RNA-seq or splicing microarray data. The output is a ranking of splicing factors that putatively regulate a condition. This ranking can be augmented with RBPs’ differential expression.

### Integrating and mapping CLIP experiments to splicing regions

There are four main variants for genome-wide CLIP experiments: (i) HITS-CLIP (high-throughput sequencing of RNA isolated by crosslinking immunoprecipitation [37], (ii) PAR-CLIP (photoactivatable ribonucleoside-enhanced cross-linking immunoprecipitation) [38], (iii) iCLIP (individual-nucleotide resolution CLIP) [39] and (iv) eCLIP (enhanced CLIP) [40]. In this manuscript, we will use CLIP experiments as a common name for HITS-CLIP, PAR-CLIP, iCLIP and eCLIP. Either of these techniques is valid to uncover RBPs’ binding sites.

**Fig. 4:**
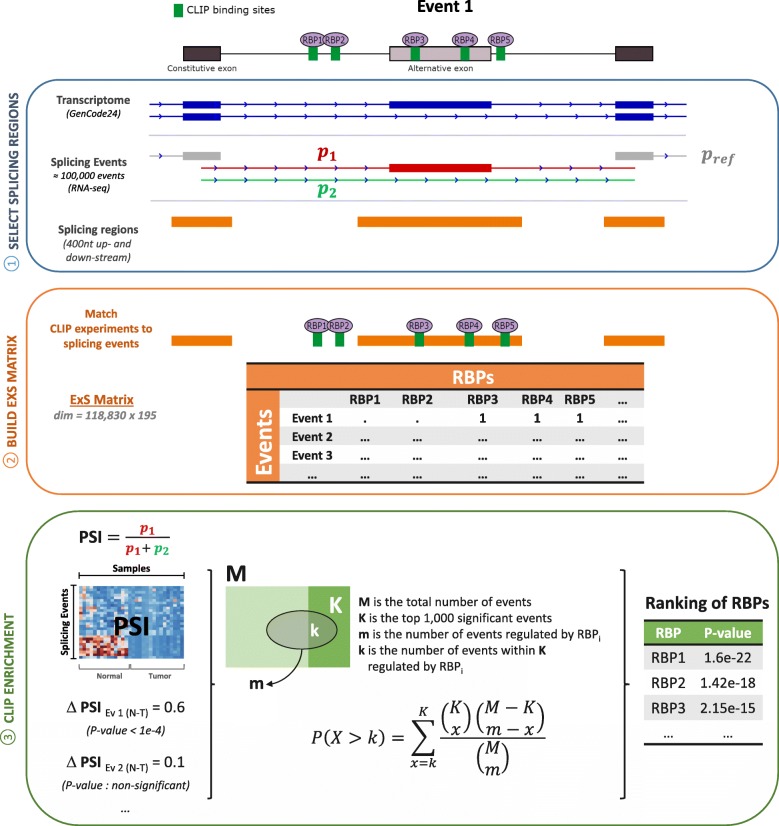
Overview of pipeline to predict splicing factors using CLIP and splicing. A toy example with a cassette exon is shown. 1) Selecting splicing regions: The cassette exon has two isoforms which give rise to Path 1 (p_1_), Path 2 (p_2_) and reference (p_ref_). Splicing regions (typically 300–400 nt upstream and down- stream the AS events [26]) are represented in orange. 2) The ExS matrix (Events x Splicing factors) is built by mapping RBPs against the splicing regions. 3) The Percent Spliced-In (PSI) of all the events is estimated from RNA-seq or microarrays and a Fisher’s exact test enrichment is performed to get a ranking of RBPs according CLIP binding sites

To relate CLIP experiments and splicing regions, we first identified all the potential splicing events for transcriptome GenCode v24 (Fig. 4, Panel 1). For each splicing event, Event Pointer returns the event type and the sub regions of the transcriptome that build up the splicing event. These sub regions are the alternative paths (*p*_*1*_ and *p*_*2*_) that form the event and a common region to both paths (*p*_*ref*_). For example, in a cassette event, the paths are: (*p*_*1*_) the cassette exon with their flanking junctions, (*p*_*2*_) the junction that skips the cassette exon and (p_ref_) the constitutive exons flanking the cassette. A more formal description is shown in [31].

Clip-seq experiments were downloaded from POSTAR2 (version SEPT 2018), CLIPdb (version 1.0), DoRiNA (version 2.0) and StarBase (version 2.0). The information about CLIP processing, genome reference and other characteristics was manually curated and gathered together (Additional file 1 Supplementary material S1).

In POSTAR2, after normalizing the data, they converted the HITS-CLIP, PAR-CLIP and iCLIP files to hg38. eCLIP files were directly downloaded in hg38 format from the ENCODE data portal (https://www.encodeproject.org/). CLIP experiments of CLIPdb, DoRiNA and StarBase include different genome versions and different species. We focused specifically on human and mouse. We converted all the different genome versions (hg18, hg19, mm8, mm9 and mm10) to hg38 with the aid of the liftOver tool and the Bioconductor packages: rtracklayer [41] and Genomic Ranges [42].

All the CLIP files, previously converted to hg38, were mapped against adjacent splicing regions. For each RBP, we summarized its CLIP experiments into a single dataset following an inclusive criterion: if a binding site is annotated to any CLIP experiment, it is considered as a putative regulation. We took POSTAR2 as the reference database, since it includes the largest number of RBPs (Fig. 1a). For each RBP not included in POSTAR2, we consider the binding sites in the other three databases. As a result of this mapping, we got a binary matrix (named ExS, Events x Splicing factors) relating splicing events with RBPs (Fig. 4, Panel 2).

We then identified the genomic regions where RBPs bind to regulate splicing. These regions will be named as splicing regions. Splicing regions are located in the neighborhood of the splicing events (300–400 nt) [26]. We selected a window of 400 nt before and after the loci of the alternative paths to be the splicing regions (Fig. 4, Panel 2). We mapped the splicing regions against the CLIP peaks of RBPs and stored it in an indicial sparse matrix **ExS** (Events x Splicing factors). Each element denotes whether the splicing factor *j* binds to the event *i* as follows:

$$ {exs^{\ast}}_{ij}=\left\{\begin{array}{c}1,\mathrm{any}\ \mathrm{of}\ \mathrm{the}\ \mathrm{splicing}\ \mathrm{factor}\ j\ \mathrm{peaks}\ \mathrm{match}\ \mathrm{to}\ \mathrm{loci}\ \mathrm{in}\ \mathrm{the}\ \mathrm{event}\ i\\ {}0,\mathrm{NONE}\ \mathrm{of}\ \mathrm{splicing}\ \mathrm{factor}\ j\  peaks\ \mathrm{match}\ \mathrm{to}\ \mathrm{loci}\ \mathrm{in}\ \mathrm{the}\ \mathrm{event}\ \mathrm{i}\ \end{array}\right. $$

**ExS** matrix provides a convenient and efficient way to compute the overrepresentation of RBPs in the differentially spliced loci for a given experiment.

### Predicting context-specific splicing factors

The second pipeline is the analysis of RNA-seq experiments to decipher which are the splicing factors that regulate splicing (Fig. 4, Panel 3). This approach assumes that the driving splicing factors must bind to differentially spliced events. Changes in splicing events are usually measured by the Percent Spliced-In (PSI). PSI is defined as the relative expression of one path of the event against the expression of the reference, as follows:

$$ PSI=\frac{p_1}{p_1+{p}_2}=\frac{p_1}{p_{ref}} $$

where *p*_*1*_ and *p*_*2*_ are the expression of the two alternative paths of a splicing event and *p*_*ref*_ is the expression of the nearest common region of the alternative paths. An expression filter is set to remove lowly expressed events and events that only express one path –in which there is not alternative splicing. In this filter, the three paths are required to express at least quantile 0.1 in 75% samples.

The RNA-seq data are processed to get the transcript expression using Kallisto with the same reference transcriptome as used in the construction of the **ExS** matrix. The PSI for all the events in the transcriptome (118,830 in GenCode v24) is estimated using Event Pointer. A statistical significance is assessed to each event following the standard pipeline of Event Pointer using the test based on the PSI (one of the paths must decrease and the other increase). The process for microarrays is described in [8].

Using a threshold on the *p*-value or on the false discovery rate is possible to select a number of events differentially spliced. In our case, the top 1000 events with most significant Event Pointer *p*-value were selected. The group of differentially spliced events is used to perform a Fisher’s exact test for all the RBPs in the database with the aid of the CLIP experiments stored in the **ExS** matrix, as follows:

$$ P\left(X>k\right)={\sum}_{x=k}^K\frac{\left(\begin{array}{c}K\\ {}x\end{array}\right)\left(\begin{array}{c}M-K\\ {}m-x\end{array}\right)}{\left(\begin{array}{c}M\\ {}m\end{array}\right)}, $$

where *M* is the total number of events, *K* is 1000 –the number of selected events-, *m* is the number of events regulated by RBP_i_ and *k* is the number of events within **K** regulated by RBP_i_ (Fig. 4, Panel 3). RBPs are ranked according *p*-value of the CLIP enrichment test.

RBPs’ gene expression can be used as an independent source of information to augment the CLIP enrichment test. The standard pipeline of limma [43] was used to get the differentially expressed RBPs and the corresponding *p*-value.

These 2 *p*-values (CLIP enrichment and gene expression) can be summarized in different ways: Fisher and Stouffer methods [44, 45], summing up the *p*-values and correct the sum by the Irvin-Hall distribution, etc. In this work, the *p*-values have not been summarized since the proposed gold-standard experiments directly knock-down a RBP. The expression changes of the knock-down genes are strongly significant due to the efficiency of the siRNAs. Therefore, summarizing the *p*-values in our experiments would return an optimistic view of the pipeline, as it would benefit only those RBPs that change their expression. We simply set a loose filter on the RBPs that were differentially expressed (limma *p*-value < 0.05; |log2 FC| > 0.58).

## Additional files

Additional file 1: **Supplementary material S1.** CLIP information of files used in the main paper. **Supplementary material S2.** CLIP experiments of RNA-binding proteins (RBPs) integrated in this work (*n* = 937). CLIP experiments were downloaded from three CLIP databases: POSTAR2 [P], CLIPdb [C], DoRiNA [D] and StarBase [S]. For each RBP, the table includes: number of experiments (#Ex), database(s) that include the RBP (DDBB) and number of splicing events where the RBP binds after integrating all CLIP experiments. (XLSX 9261 kb)

Additional file 2: **Figure S3. (Right-hand side)** Pearson correlation coefficient heatmap representing the similarity of RNA-binding protein binding sites in splicing events. Red and blue indicate higher and lower correlation, respectively. **(Left-hand side)** Information of the four experiments analyzed in this manuscript (Table 1). KD-SRSF1 (green), KD-TARDBP (blue), KD-TAF15 (yellow), and KD-FUS (red). Each experiment shows two color lines: Expression *p*-value <1e-3 (dark color) and CLIP *p*-value <1e4 (light color). The CLIP *p*-value of KD-TAF15 (light yellow) is empty because no RBP passed the CLIP *p*-value threshold (1e3).The names of the four knock-down RBPs are highlighted with yellow squares. Remarkably, RBPs that belong to similar families tend to cluster together(e.g.IGF2BP-X-METTL-X,CPSF-X,SF3-X,TRNC-X,YTHD-X). (PDF 286 kb)

## Data Availability

The source code, databases and a tutorial are available in the GitLab repository, https://gitlab.com/fcarazo.m/sfprediction.

## Abbreviations

AS: Alternative splicing
CLIP: Cross-linking immunoprecipitation
ExS: Event x splicing factor
PSI: Percent splice-In
PWM: Position weight matrix
RBP: RNA-binding protein
SF: Splicing factors
