# Supplementary material for: Integration of CLIP experiments of RNA-binding proteins: a novel approach to predict context-dependent splicing factors from transcriptomic data
Source: BMC Genomics. 2019 Jun 25;20:521. doi: 10.1186/s12864-019-5900-1 (PMC6592009; doi:10.1186/s12864-019-5900-1)
Supplement: Supplementary file 2 — Figure S3. (Right-hand side) Pearson correlation coefficient heatmap representing the similarity of RNA-binding protein binding sites in splicing events. Red and blue indicate higher and lower correlation, respectively. (Left-hand side) Information of the four experiments analyzed in this manuscript (Table 1). KD-SRSF1 (green), KD-TARDBP (blue), KD-TAF15 (yellow), and KD-FUS (red). Each experiment shows two color lines: Expression p-value <1e-3 (dark color) and CLIP p-value <1e4 (light color). The CLIP p-value of KD-TAF15 (light yellow) is empty because no RBP passed the CLIP p-value threshold (1e3).The names of the four knock-down RBPs are highlighted with yellow squares. Remarkably, RBPs that belong to similar families tend to cluster together(e.g.IGF2BP-X-METTL-X,CPSF-X,SF3-X,TRNC-X,YTHD-X). (PDF 286 kb) [file 12864_2019_5900_MOESM2_ESM.pdf]

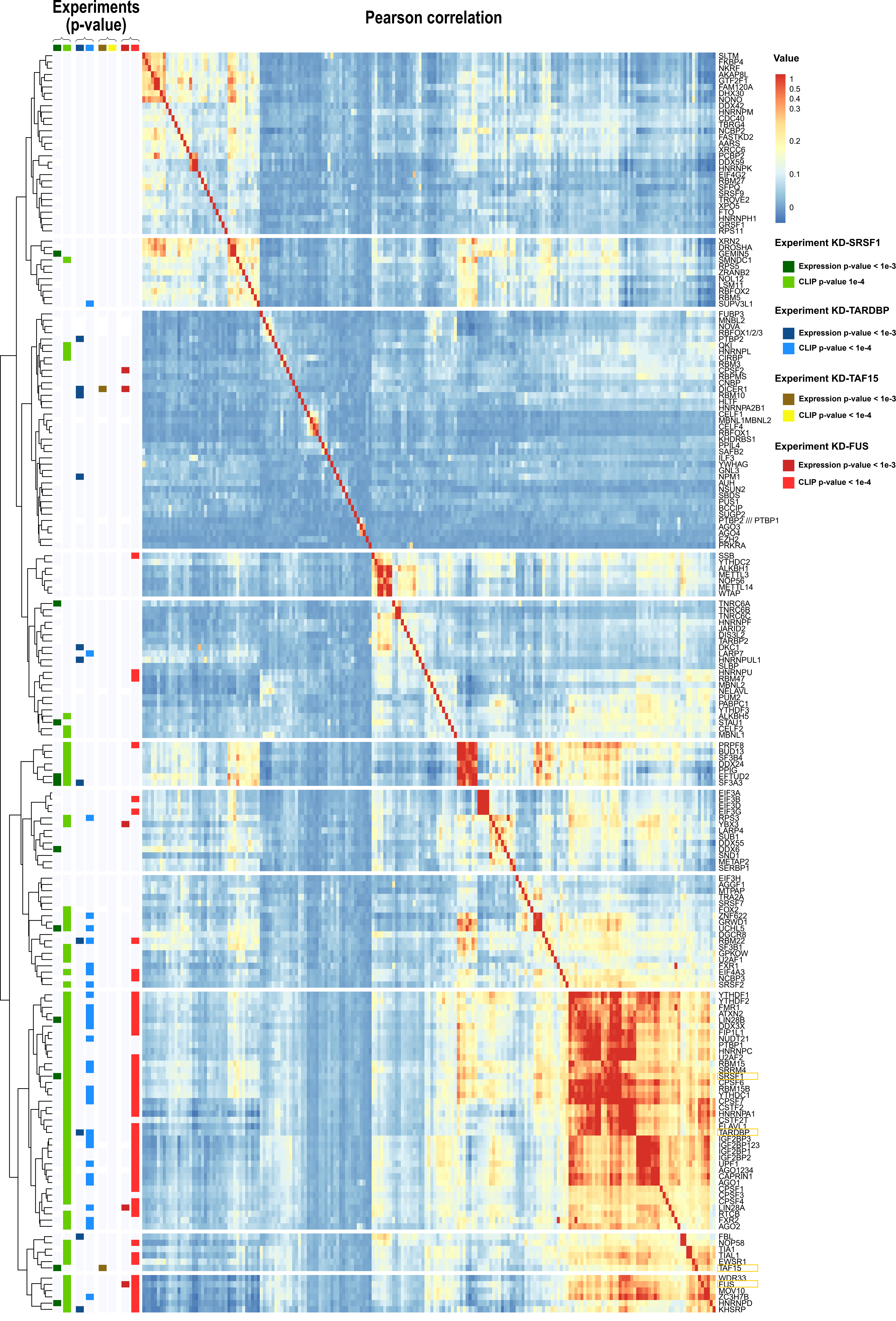

*Figure S3: **(Right-hand side)** Pearson correlation coefficient heatmap representing the similarity of RNA-binding protein binding sites in splicing events. Red and blue indicate higher and lower correlation, respectively. **(Left-hand side)** Information of the four experiments analyzed in this manuscript (Table 1). KD-SRSF1 (green), KD-TARDBP (blue), KD-TAF15 (yellow), and KD-FUS (red). Each experiment shows two color lines: Expression p-value < 1e-3 (dark color) and CLIP p-value < 1e-4 (light color). The CLIP p-value of KD-TAF15 (light yellow) is empty because no RBP passed the CLIP p-value threshold (1e-3). The names of the four knock-down RBPs are highlighted with yellow squares. Remarkably, RBPs that belong to similar families tend to cluster together (e.g. IGF2BP-X, EIF3-X, METTL-X, CPSF-X, SF3-X, TRNC-X, YTHD-X).*
